# Supplementary material for: miR-154-5p Is a Novel Endogenous Ligand for TLR7 Inducing Microglial Activation and Neuronal Injury
Source: Cells. 2024 Feb 26;13(5):407. doi: 10.3390/cells13050407 (PMC10930870; doi:10.3390/cells13050407)
Supplement: Supplementary file 1 [file cells-13-00407-s001.zip › cells-2868599-supplementary.pdf]

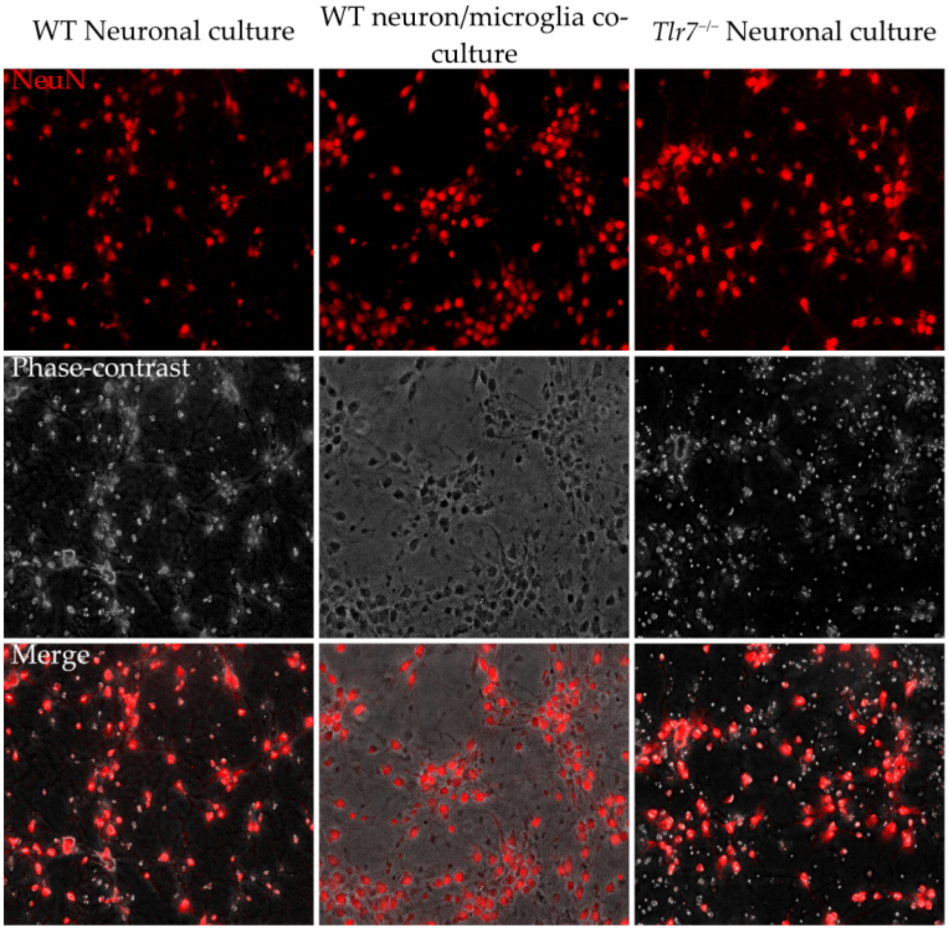

Figure S1. Representative images of enriched cortical neuron cultures and co-cultures containing neurons and microglia. Enriched cortical neuron cultures (wild-type, WT, C56BL/6 and *Tlr7*-/-) and WT co-cultures containing neurons and microglia were immunostained with NeuN (red fluorescence) or analyzed by phase-contrast microscopy, as indicated. 20× magnification.
